# Laparoscopic Distal Pancreatectomy with Splenic Conservation: An Operation without Increased Morbidity

**DOI:** 10.1155/2009/846340

**Published:** 2009-12-16

**Authors:** Peter Nau, W. Scott Melvin, Vimal K. Narula, P. Mark Bloomston, E. Christopher Ellison, Peter Muscarella

**Affiliations:** Department of Surgery, The Ohio State University Medical Center, Columbus, OH 43210-1228, USA

## Abstract

*Objectives*. The advent of minimally invasive techniques was marked by a paradigm shift towards the use of laparoscopy for benign distal pancreatic masses. Herein we describe one center's experience with laparoscopic distal pancreatectomy. *Methods*. A retrospective chart review was performed for all distal pancreatectomies completed laparoscopically from 1999 to 2009. Outcomes from those cases completed with a concurrent splenectomy were compared to the spleen-preserving procedures. *Results*. Twenty-four patients underwent laparoscopic distal pancreatectomy. Seven had spleen-conserving operations. There was no difference in the mean estimated blood loss (316 versus 285 mL, *P* = .5) or operative time (179 versus 170 minutes, *P* = .9). The mean tumor size was not significantly different (3.1 versus 2.2 cm, *P* = .9). There was no difference in the average hospital stay (7.1 versus 7.0 days, *P* = .7). Complications in the spleen-preserving group included one iatrogenic colon injury, two pancreatic fistulas, and two cases of iatrogenic diabetes. In the splenectomy group, two developed respiratory failure, three acquired iatrogenic diabetes, and two suffered pancreatic fistulas (71% versus 41%, *P* = .4). *Conclusions*. The laparoscopic distal pancreatectomy is a safe operation with a low morbidity. Splenic conservation does not significantly increase the morbidity of the procedure.

## 1. Background

Minimally invasive approaches to surgical procedures have become commonplace in general surgery beginning with Dubois's seminal article describing a laparoscopic dissection of the gallbladder in 1990 [1]. While the laparoscopic distal pancreatectomy requires advanced laparoscopic skills, it is a well-described technique to approach a pancreatic tail mass [2–5]. To date, there is a disparity in the outcomes that different groups have reported in reference to the operative details and postoperative complications [6–10]. Consequently, it is difficult to adequately characterize the morbidities and outcomes of this approach.

There are conflicting reports of the morbidity of distal pancreatectomy with concomitant splenectomy. A comprehensive review by Holdsworth describes low complication rates when a splenectomy is included with the distal pancreas resection [11]. Conversely, Shoup et al. identified a trend towards increased infectious complications and length of stay with the addition of a splenectomy [12]. Despite this incongruity, splenic salvage has become the standard of care unless otherwise contraindicated in patients with benign lesions. 

There are two distinct approaches to conserve the spleen during the dissection of the distal pancreas. The classic design is to identify, isolate, and preserve the splenic artery and vein [13]. Alternatively, Warshaw described spleen-preserving techniques during which the splenic artery and vein are ligated with the pancreas, and perfusion of the spleen is maintained by the short gastric vessels [14]. Both are accepted as appropriate techniques to address a mass in the tail of the pancreas.

A review of literature highlights the inconsistencies of the outcomes, both during the procedure and postoperatively. While most authors feel that the operation is safe, the reported complications are inconsistent in breadth and severity [7, 15]. For instance, a persistent fistula from the pancreatic remnant is the most common adverse event. However, it is difficult to characterize this complication because it is reported to occur anywhere from 4% to 50% of the time and is ambiguous in its definition [3, 16]. Additionally, to date, there is little discussion referencing the incidence of endocrine insufficiency following pancreatic tail resection. Furthermore, operative times and associated intraoperative blood loss vary widely depending on the source. 

The purpose of this study was to outline one institution's experience with the laparoscopic distal pancreatectomy without splenectomy (LDP) and laparoscopic distal pancreatectomy with splenectomy (LDPS), and to further describe the associated morbidities.

## 2. Methods

Patients enrolled in this study were identified from a pancreatic database by their ICD-9 code as having pathology of distal pancreas from the period of July 1999 until July 2008. All information was collected retrospectively through the review of hospital and clinic charts. Demographic, procedural, and postoperative data were documented. Pancreatic fistulas were defined as the persistence of amylase-rich fluid three times the upper normal serum value from an intraoperatively placed drain three days postoperatively [17]. 

**Figure 1:**
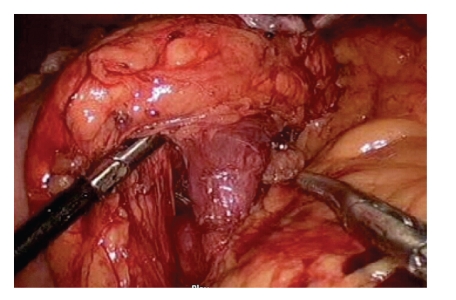
Identification and isolation of splenic vein at the dorsal aspect of the pancreas.

**Figure 2:**
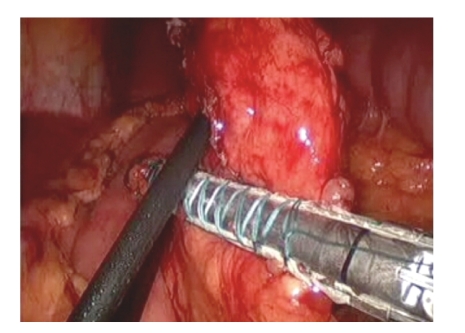
Transection of the pancreas with a linear stapling device using a bioabsorbable staple line reinforcement system.

**Figure 3:**
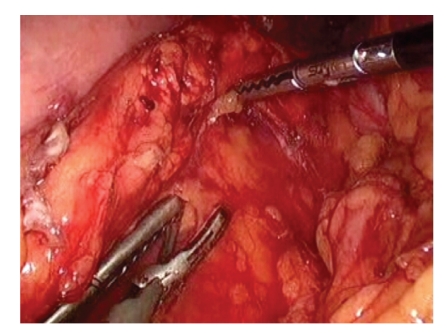
Dissection of the pancreas off the splenic vessels for spleen preservation.

The technique for LDP has been described elsewhere [13]. In short, after establishing laparoscopic access, the pancreas is exposed through the division of the lesser sac. The splenic attachments and the short gastric vessels are divided. The mass is identified and its resectability is verified. The splenic artery and vein are then identified and isolated using the Maryland dissectors and a harmonic scalpel (Figure 1). The pancreas is transected with a single application of a linear stapling device with a bioabsorbable staple line reinforcement system (W.L. GORE, Flagstaff, Ariz, USA) (Figure 2). The splenic vessels are dissected from the pancreas to the splenic hilum (Figure 3). The specimen is then placed into an endoscopic bag and delivered from the abdomen. Peritoneal drains are placed near the transected surface of the pancreas and brought out of the abdomen through the 5-mm port sites.

The LDPS is completed in a similar manner. The pancreas is exposed, and the mass is identified according to the technique used in the spleen-preserving procedure. The splenic artery and vein are identified and individually divided with 2.5 mm linear stapling devices. The pancreas is then transected. The remainder of the procedure mirrors that of the LDP protocol.

For those variables that satisfied the normality and equal variance assumptions, Student's *t*-tests were performed. Non-parametric data were analyzed with the Mann-Whitney test. Binary variables were assessed with Fisher's Exact test. Statistical significance was defined as *P* < .05. Permission from the Ohio State University Medical Center Institutional Review Board (IRB) was obtained prior to beginning the study. HIPPA compliance was maintained throughout the data collection process.

## 3. Results

A total of 24 distal pancreatectomies was completed laparoscopically. Demographic data for both groups are demonstrated in Table 1. Fifty-seven percent of patients undergoing LDP and 41% of those with an LDPS had prior intraabdominal surgery. The majority of patients presented with abdominal pain and a work-up with computed tomography (CT) scanning that identified the pancreatic lesion (42%). Thirty-three percent had pancreatic masses discovered incidentally during imaging for reasons unrelated to the final diagnosis. Additional 25% of the patients presented with symptomatic neuroendocrine tumors.

Operative data are shown in Table 2. It is noteworthy that there were no differences in the mean operative times, blood loss, or necessity of transfusion. Seven spleen-conserving operations were completed. In four cases, the tumor size and appearance were concerned for malignancy, and a splenectomy was chosen for an appropriate oncologic resection. Nine patients had anatomical variations that necessitated a splenectomy, including intrapancreatic splenic vasculature (2), adhesions secondary to pancreatitis (1), and mass location in relation to the spleen and its blood supply (6). Additionally, the splenic vessels were traumatized in one patient during the course of dissection, necessitating a splenectomy. There were no conversions to open procedures. The mean postoperative length of stay for both groups was seven days. The 30-day mortality was zero for both groups.

The mean tumor size was 3.1 cm and 2.2 cm for the LDPS and LDP groups, respectively, (*P*-value = .9). In 83% of the cases, the mass was palpable. Intraoperative ultrasound was successfully used to localize a nonpalpable tumor in 3 cases. The final pathologic diagnoses are depicted in Table 3. An R0 resection was obtained in all of the LDP operations and 80% of the LDPS group. There were two R1 resections, and in only one case, an R2 resection was completed. 

In the spleen-conserving cohort, there were five complications. One patient required reoperation on postoperative day four for hypotension and bloody drainage from intraoperatively placed drains. Intraoperative findings included bleeding from the omentum and a contained iatrogenic perforation of the transverse colon. The hemorrhagic omentum was excised, and the colon defect was repaired primarily. Two patients developed Grade A pancreatic fistulas that responded to conservative management. Two patients developed iatrogenic diabetes and were discharged on subcutaneous insulin regimens for the maintenance of normoglycemia. 

There were eight complications in the LDPS group. Two patients were reintubated for respiratory distress. One patient was subsequently noted to have a partial small bowel obstruction on an abdominal CT. This was treated conservatively. The patient was extubated four days later and discharged on postoperative day number 21 tolerating a regular diet with normal bowel function. The second patient was noted to have an intraabdominal fluid collection during a work up for tachypnea and tachycardia. This was addressed laparoscopically on postoperative day four. Afterwards, the patient remained intubated secondary to poorly managed asthma. The patient was extubated three days later and discharged on postoperative day twelve from the initial operation. Three patients developed pancreatic fistulas. These were Grade A fistulas that were managed conservatively and required no additional intervention. Three patients developed iatrogenic diabetes and were discharged home on subcutaneous insulin regimens. 

## 4. Discussion

The necessity of a distal pancreatectomy with splenic preservation has been extensively debated in literature. The infectious risk posed to the asplenic patient is most often used as the argument for an LDP. However, in a review of almost 6000 surgical cases, Holdsworth et al. noted that the incidence of infection after splenectomy was 0.9% with a mortality rate of only 0.8% [11]. Additionally, many infections were unrelated to the asplenic state and were uncommon outside the pediatric population. Notwithstanding, the preferred operation for patients with benign pancreatic disease is the one that leaves the spleen in situ when technically feasible. At the Ohio State University Medical Center, the splenic artery and vein are routinely identified and spared during a distal pancreatectomy without splenectomy. The technique described by Warshaw is eschewed due to the increased risk of spleen infarction and abscess formation postoperatively. To date, there have been no ischemic complications in patients that underwent splenic salvage in this series. Moreover, this is the first study to show no differences in the morbidities and operating times between the LDP and LDPS groups.

For this cohort of patients, there were seven LDP cases. In 29%, the rate of splenic salvage is comparable to published rates [10, 15]. In this series, proximity of the mass to the spleen and the orientation of the splenic artery and vein dictated the intraoperative decision for splenectomy, 38% of the time. In additional four cases, the tumor size and appearance were concerned for malignancy, and a splenectomy was chosen for an appropriate oncologic resection. In one patient the splenic vessels were traumatized during the course of dissection necessitating a splenectomy. There were no differences in operative morbidities between the two groups. The blood loss was virtually identical for the LDPS (316 mL) and the LDP (285 mL) groups (*P*-value = .5). Furthermore, the operative time was similar between the LDPS and LDP cohorts (179 minutes versus 170 minutes, *P*-value = .99). Lastly, there was no significant difference in the size of the masses between groups (*P*-value = .9). 

Five patients developed Grade A pancreatic fistulas (21%). All were managed conservatively and resolved within six weeks of the operation. The other most common adverse event noted was the development of postoperative endocrine insufficiency in five patients (21%). Interestingly, three of the five patients who developed diabetes initially presented with symptoms of hypoglycemia and had a final pathologic diagnosis of an insulinoma. 

In a paper by Pandey et al., the anatomical variation of the splenic artery is described as having the classic suprapancreatic orientation only, 74% of the time [18]. They also noted that the artery may divide into more than two terminal branches in 34% of the cases prior to encountering the hilum of the spleen. It is this variability that makes isolation and preservation of the splenic vasculature complex. Many reports on LDP have used the Warshaw protocol which does not take into account this variability, as the blood supply is not reliant upon those vessels for survival. This technique is not without risks as it is associated with postoperative hypoperfusion of the spleen with infarction, potential infection, and the necessity of reexploration [10, 13]. The rate of splenic salvage in this series was comparable to previous studies notwithstanding the vessel-conserving approach. It is important to note that 53% of LDPS were performed secondary to the orientation of the splenic vasculature and the adherence to a vessel-preserving technique. It is foreseeable that fewer splenectomies would have been performed with the application of the Warshaw technique.

Historically, the most common complication following LDP and LDPS has been a persistent leak from the pancreatic remnant. Pryor et al. reported a 50% rate of pancreatic fistula formation [10]. Unlike most, however, they categorized their leaks based on intervention and severity. Other studies have noted leak rates anywhere from 4%–52%, often without a clearly stated definition [9]. This had been a limitation of literature in the past. However, a new international standard for pancreatic fistulas has been drafted [17]. These guidelines parallel the definition that we employed at our institution prior to the release of this consensus statement. These criteria should facilitate the characterization of this complication, specifically the incidence and expected morbidity. 

There are also inconsistencies in the other morbidities to which a patient is exposed in an LDP versus LDPS. Benoist noted that their pancreatic fistula rate, intraabdominal abscess formation, and length of stay were all greater in the LDP group [19]. Conversely, most data support the LDP as a less morbid operation with a decrease in the length of stay and rate of infection [12, 20, 21]. In this retrospective review, there were no differences noted in the morbidities to which the LDP and LDPS groups were exposed. As proficiency in laparoscopy progresses, it would be expected that the perioperative and postoperative outcomes should become increasingly homogeneous.

## 5. Conclusion

A laparoscopic approach to a pancreatic tail mass is both feasible and safe. This is the first study to identify no difference between the perioperative characteristics of LDP and LDPS. Previous reports have avoided dissection of the splenic vasculature due to the expected increase in operative time and blood loss. This was not the case in this cohort. Maintenance of the anatomical perfusion of the spleen during laparoscopic spleen-preserving distal pancreatectomy is an invaluable tool for the laparoscopic surgeon. 

## Figures and Tables

**Table 1:** Patient demographics.

Parameter	Splenectomy	Spleen conserving	Total
Number patients	17	7	24
Gender (m/f)	7/10	1/6	8/16
Mean patient age (range)	51 (23–78)	51 (36–61)	51 (23–78)
Mean ASA score (range)	2.8 (2-3)	2.9 (2-3)	2.8 (2-3)
Mean body mass index (range)	29.3 (24.2–34.8)	32.7 (25.2–45.3)	30.3 (24.2–45.3)

**Table 2:** Operative characteristics.

Parameter	Splenectomy	Spleen conserving	*P*-value
Mean operativetime – minutes (range)	179 (114 – 326)	170 (74 – 288)	0.9
Mean tumor size, cm (range)	3.1 (1.6–6.0)	2.2 (0.9–4.0)	0.9
Mean bloodloss, mL(range)	316 (50–1200)	285 (50–1200)	0.5
Number of patients transfused	2	1	1.0
Postop Length of stay, days (range)	7.0 (4–22)	7.1 (2–15)	0.4
30-day mortality	0	0	1.0

**Table 3:** Tumor characteristics.

Pathology	Splenectomy	Spleen conserving	Combined
Adenocarcinoma	1 (5%)	0 (0%)	1 (5%)
Neuroendocrine	4 (17%)	4 (17%)	8 (33%)
IPMN	1 (4%)	1 (4%)	2 (8%)
Serous microcystic adenoma	4 (17%)	1 (4%)	5 (21%)
Mucinous cystadenoma	2 (8%)	1 (4%)	3 (13%)
Other	5 (21%)	0 (0%)	5 (21%)
Total	17 (71%)	7 (29%)	24
